# Ten Years HIV Free: An Interview with “The Berlin Patient,” Timothy Ray Brown

**DOI:** 10.20411/pai.v2i3.226

**Published:** 2017-11-20

**Authors:** Michael M. Lederman, Earl Pike

**Affiliations:** 1 Case Western Reserve University School of Medicine, Cleveland, Ohio; 2 Former CEO of AIDS Taskforce of Greater Cleveland, Cleveland, Ohio

**Keywords:** HIV, immunization, antiretroviral

## INTRODUCTION

It was just about 10 years ago that Timothy Ray Brown was cured of HIV infection by receiving bone marrow stem cell transplantations from an HLA matched donor who also was homozygous for the CCR5 delta 32 mutation that renders immune cells resistant to infection by most HIV viruses. During an October 2017 visit to Cleveland to help launch a number of clinical trials targeting the Cure, Timothy sat down with *Pathogens and Immunity* editor Michael Lederman and community activist Earl Pike to talk about his experiences.

### Pathogens and Immunity

Have you always seen yourself as an activist? Were you an activist before you were diagnosed?

### Timothy Ray Brown

Before I left the United States I was looking for ways to become politically active somehow so I started going to Act Up Seattle meetings. That was in 1989 or '90. Not long after that I moved to Barcelona and was teaching English. I was probably infected in Barcelona. I had learned some German at that point and I prepared to study in Berlin. At that time I wasn't involved politically at all. I chose political science as my major; sociology was part of that. I learned about the German government and I learned to speak, read, and write German very well.

When I moved back to Berlin I was staying at a friend's place. He had taken a trip to Reykjavík [Iceland]. I was staying in his [Berlin] apartment for about a week and I got a really high fever and I was on the couch, covered up. I was diagnosed in 1995 at a state-operated tropical medicine clinic. The doctor who gave me my diagnosis assumed that I had had HIV longer because my T cell count was down to 300 or something like that. She said, “You need to start medication, immediately.” I was scared of AZT (azidothymidine).

### PAI

Is that what they offered you in '95? You probably heard a lot of bad stuff about AZT.

### TRB

Yeah. I didn't want AZT. She said, “I'm going to give you a very low dose of it.” So, she gave me a very low dose and I took that for probably about a year, but it could have been shorter. She was going off to Africa to work, so I asked her, “Who do you suggest I go to?” And she suggested Dr. [Heiko] Jessen, so I started seeing Dr. Jessen, who took me off AZT almost immediately. I started seeing his brother and saw him until I found out I had leukemia in mid-2006.

### PAI

But by then you were on combination therapy?

### TRB

Yeah …

### PAI

You've had at least two scary diagnoses, first, the diagnosis of HIV in '95, that was just at the beginning of the HAART (highly active antiretroviral therapy) era. We were just learning about combination therapy and how lifesaving it was and people probably didn't know that for sure by '95. And then a diagnosis of acute leukemia, which would have been 10 or 11 years ago in Berlin. Can you tell us a little about how you felt about those two very different diagnoses?

### TRB

After my diagnosis of being HIV positive I remember a friend of mine said, “You know we only have about two more years to live.” That was a huge shock.

### PAI

When you got that diagnosis of leukemia, how did you take it?

### TRB

Not as hard as my partner, Michael. My partner wailed. He was really upset. I was more unemotional about it than he was. I was scared. The oncologist that I went to told me that I had to go through chemotherapy. I thought, OK, one round of chemo and then it may be over. Then I found out it was going to be four rounds of chemo. I had sepsis during the third round and I thought, “I don't want to do this anymore.” I asked my doctor what I should do, and he said, “Go someplace you really like for a vacation.” And so I chose to go to Genoa in Italy.

### PAI

We know the degree to which people internalize the stigma and shame of an HIV diagnosis. Did you find yourself still internalizing some of that?

### TRB

In Berlin I didn't really feel stigmatized. Right after my diagnosis, I told my bosses, the two owners and managers of a café at which I worked. I told all of my coworkers as well. I didn't go around the restaurant saying, “Hey I'm HIV positive,” but they were supportive. I think Berlin is, or was at the time, a very open place. I don't know if it still is; I haven't lived there since 2010. I felt very little stigma. I understand how much stigma there was here and, going to Africa, how much stigma is there. I understand it, but I didn't feel it myself.

### PAI

When they told you you were cured of HIV, when did you start believing it? Did you believe that you were cured right away or was that a process?

### TRB

It was kind of a process. I noticed it physically when I went back to the gym. I had had the wasting syndrome with HIV. After the first transplant, I went back to the gym and I noticed I was gaining muscle weight and I was looking better. I don't think I really believed I was cured until Gero Hütter's article [detailing my cure] was published by the *New England Journal of Medicine* [1]. They had originally rejected the article twice, but at a meeting (where my story was presented) they asked everyone present, “Do you think that this patient has been cured of HIV?” And everyone raised their hands. A year later a journalist from the *Washington Post* said, “OK, I think I'm going to publish this.” So he did and the *New York Times* didn't want to be beaten to the punch so they published it and then the *New England Journal of Medicine* came and said, “We've reconsidered it. We're going to publish it.”

### PAI

You have had some difficulty walking after your second bone marrow transplant. What do you think was responsible for your gait troubles?

### TRB

At one point I was delirious and they didn't know why and they were trying to figure it out. So, they thought I might have leukemia in my brain. So they did a brain biopsy and they, of course, looked for HIV. They didn't find either, which was very good news because it meant that I didn't have HIV, at least in that tissue sample that they took in one of the main reservoirs for HIV. So, that was kind of the proof that it was gone, probably from my entire body. I've had countless biopsies, lots of colonoscopies and a really intense colonoscopy that was performed by [HIV researcher] Tim Schacker who also did a biopsy of a lymph node.

My major neurologic thing is balance. That was probably from the air bubble that was left in my brain after the biopsy. They corrected the air bubble, but it still left the damage. I didn't know what was causing that for a long time. I thought it had to do with the radiation or with the chemo, but Gero told my mother that he thought it had to do with the air bubble. I went to physical therapy. The first thing I went there for was my arm, my shoulder. Then I continued to go to a second physical therapist to work on my balance.

[HIV researcher] Steve Deeks came up to me after an announcement about a SCOPE study that had these two patients that they thought might be cured, but the medical team wasn't really sure. He said, “We want to inject their blood into humanized mice and see if the HIV grows in them and we would also like to do it with you.” My former business partner said, “No, no way. You've done enough.” Later I broke off ties with my former business partner and I went to Steve and said, “I'll do it.” We made arrangements for me to go to San Francisco. They wanted to make sure I was healthy enough to do a leukapheresis. That was the beginning of July. They did the leukapheresis at the end of July. [HIV Researcher] Tim Henrich was there and he said, “We are basically looking for the definitive reason why you are cured. We are looking for CD32a+ cells” (cells that may be enriched for the presence of latent HIV).

### PAI

That's a great experiment. We don't know if there is any infection-competent virus left in you. For you it's almost irrelevant because there's no place for these guys to rekindle infection. Your immune system is repopulated with HIV-resistant cells. It's great that you let them take some cells out. If there are any infection-competent viruses in you they may not be in circulation.

### TRB

I take PREP now. And probably the fact that I take PREP might keep them from reproducing.

### PAI

Someone recently said that your being cured was a little like Neil Armstrong stepping on the moon [2]. It proved we could do it, but it didn't prove that we could scale it up. What do you think about that?

### TRB

I was just in Cape Town, South Africa and went to one of the Desmond Tutu Foundation clinics. I got a tour of it and they asked me to speak in front of the waiting room. It was important to me and to the directors there that I didn't give false hope, and I wanted to make sure that people understood that my case was just a case in point. That HIV can be cured and you don't want to do it the way I did it.

### PAI

We've heard you speak before about your experiences with battling leukemia and stem-cell transplantation and graft versus host and we've seen people and cared for people who are undergoing this kind of intensive chemotherapy and stem-cell transplantation for leukemia. It's pretty scary. If someone who was HIV positive and developed leukemia were to ask if you would recommend undergoing a similar thing, what would you say?

### TRB

I had a similar situation with a guy I met at (another) University. He had AML (Acute myeloid leukemia) like me along with HIV and was about to undergo a stem-cell transplant. I basically said, “Make sure to try to find a donor who is CCR5 delta32.” They looked for one and there was something wrong with the match.

### PAI

So, it wasn't as good an HLA match …

### TRB

Yeah, they decided they didn't want to do it with that donor. They ended up getting an HLA match with a heterozygous (CCR5 delta 32 donor) and he himself was already heterozygous but the donor was not homozygous. So, they don't really know what's going to happen to him. He's undergone it and he's doing well so far. So if he survives he might be number two.

### PAI

You've had so many awesome caregivers over the years. What did individuals do that really helped you along the way? What advice would you have for physicians with a patient who is considering a treatment as intense as the treatment you've received? What did the good docs do?

### TRB

Probably the first person who comes to mind is Gero Hütter. I would say having an open mind and defying odds. We were both at this conference in Marseille, and Alain Lafeuillade kept calling Gero a genius. Gero is much too humble to allow people to call him that. He was like, “I'm not a genius. I just had a good idea.”

From the beginning it was really experimental. I was a guinea pig and it happened to work. I'm beginning to think that everything works out for a reason. I'm beginning to think that some entity, some higher power or whatever, chose me because they knew that I would be an advocate for the science to keep going.

### PAI

Some people, when they have significant medical experiences, have multiple birthdays. Do you do that as well?

### TRB

Yeah, my real one, my transplant birthday, my cure birthday would have been another one. Nobody is really sure when that is. Do you measure it by the date of the transplant or is it the day that I was probably HIV negative?

### PAI

What do you want to do now? What do you want to do for the rest of your life?

### TRB

I've thought about becoming a nurse or something like that, something to help people.

### PAI

We all get to choose our lives, but knowing that you no longer have HIV, means that you have a different kind of choice. Someday we may have that cure, and what will people living with HIV do? Being a person with HIV and then not being a person with HIV? Although you still identify with the HIV community, right?

### TRB

Yeah, yeah. My partner is HIV positive and most of the people I know are HIV positive. I'm in a group called Let's Kick A.S.S. It's “Let's Kick AIDS Survival Syndrome.” It was started by one prominent HIV activist who advocated against using crystal meth and then started again; he decided to quit taking his medication. He eventually passed away because of it. So this activist in San Francisco, Tez Anderson, he had the idea to create a group to help people who have AIDS Survival Syndrome. Since I'm not HIV positive, technically I'm just kind of an honorary member.

### PAI

The term “honorary member” seems ironic.

### TRB

I mean, I have AIDS Survival Syndrome, kind of to the extreme. I'm the only one that's been cured. That's kind of hard.

### PAI

The support group of people who have actually been cured of HIV is actually a pretty small group. Certainly in the late '90s and thereafter, so many people who survived this epidemic experienced this syndrome because of so many friends who did not. But your situation is completely different.

### TRB

I think being in Berlin during at lot of the '90s and the 2000s, I was kind of protected from all of the people dying around me because it didn't seem like too many people were dying because of AIDS in Berlin.

### PAI

One of the questions people are not sure about is whether the inflammatory activation response that we see in the setting of HIV disease, even on treatment, will go away even if HIV is completely eradicated, as it was in your case. Tim Schacker's paper about your lymph node suggests that in fact the inflammation and fibrosis of the node goes away. Are you still on any immunosuppressants?

### TRB

No, no.

### PAI

When did you stop taking them?

### TRB

Definitely before I left Berlin, which was December 2010. I think I took them for about a year. I know I quit taking them after the first transplant. After the second transplant I think I took them about the same amount of time.

### PAI

Do you have any graft versus host disease at all now?

### TRB

No, none at all.

### PAI

The match wasn't a perfect match, was it?

### TRB

No.

### PAI

When did Dr. Schacker perform your lymph node biopsy?

### TRB

February 2012.

### PAI

A year or two or more after you stopped taking your immunosuppressants. So, you didn't have a lot fibrosis, and you didn't have a lot of inflammation in the node, and you were not on any immunosuppressants that could have falsely given that impression. That's really important. Is there any part of you that worries about HIV returning?

### TRB

No, I'm confident, particularly because I'm on PREP.

### PAI

How often do you see your doctor and where are your CD4 T cells?

### TRB

I see him quarterly. The highest I've seen it, it's been 850. It's been down to like, 600-something, so it fluctuates a lot.

### PAI

What would you say to someone who asked you, “I'm HIV positive, you're cured. Is there a cure for me in my lifetime?” The question is not, “someday,” but is a cure a reasonable expectation *for me*?

### TRB

I've always said, like everyone else, I can't give an estimate for how long it would take. I've always said that it would happen in my lifetime. I'm hoping that's true, but if it's not, it's not. What does cure mean? [Researchers] Paula Cannon and Hans-Peter Kiem wrote a paper a couple of years ago and they were saying that my case is more of a very long-term remission. And I thought, “You can't take my cure away from me!”

### PAI

We think you've been cured. Whether that means that every last replication-competent virus in your body is gone, we have no idea. As before, we don't think that's the issue. The issue is that you will not relapse. All of the other attempts to eradicate have not been successful and that's not to say that there isn't going to be a scalable method somewhere down the road. It's going to be tough. It's going to take some work and all of these trials people are doing all over the world are trying to get a better handle on what it's going to take to get to that point. We don't think we can promise that to people who come to us for trials now. That wouldn't be fair or realistic. What we can promise is that we will do these trials well and learn something from them.

Do you have anything else you would like to say, or any comments for our readers?

### TRB

I think if you have not been tested for HIV, you need to get tested. You need to know your status and if you get tested (and test positive) it's no longer a death sentence. You can basically live a normal, healthy life and you are going to have fewer health problems, because if you are HIV positive you are going to need to see a doctor at least quarterly and have your health checked. That's very important for everyone, even for people who are not HIV positive. You should really get your health checked regularly. I think one day there will be a cure. I don't think it will be one particular way to get cured; nobody wants to go through what I went through. It's going to happen. If it's happened once it will happen again. Like David Baltimore said, “Nothing is impossible in medical science.”
